# Supplementary material for: All-at-once RNA folding with 3D motif prediction framed by evolutionary information
Source: Nat Methods. 2025 Oct 3;22(10):2094–106. doi: 10.1038/s41592-025-02833-w (PMC12510878; doi:10.1038/s41592-025-02833-w)
Supplement: Supplementary file 3 — Source code to run CaCoFold-R3D. Data and results. [file 41592_2025_2833_MOESM3_ESM.gz › supplemental_material/results/CaCoFold-R3D/CaCoFold-R3D_Rfam/RF02290_TtnuCD14.R2R.sto.pdf]

RF02290_TtnuCD14

5′ - U Y R A A U G A U G U U ○ U U A G U G U G U A A U C U C U C U C A U U A G A U C C U C G G A U C U A U G U A G A A A A ● U U ○ A U C C ○ U Y A C U G A
